# Measuring mental workload in assistive wearable devices: a review

**DOI:** 10.1186/s12984-021-00953-w

**Published:** 2021-11-07

**Authors:** Charlotte Marchand, Jozina B. De Graaf, Nathanaël Jarrassé

**Affiliations:** 1 CNRS, UMR 7222, ISIR / INSERM, U1150 Agathe-ISIR, Sorbonne Université, Paris, France; 2 Aix Marseille Univ, CNRS, ISM, Marseille, France

**Keywords:** Mental workload, Prosthesis, Exoskeleton

## Abstract

As wearable assistive devices, such as prostheses and exoskeletons, become increasingly sophisticated and effective, the mental workload associated with their use remains high and becomes a major challenge to their ecological use and long-term adoption. Numerous methods of measuring mental workload co-exist, making analysis of this research topic difficult. The aim of this review is to examine how mental workload resulting from the use of wearable assistive devices has been measured, in order to gain insight into the specific possibilities and limitations of this field. Literature searches were conducted in the main scientific databases and 60 articles measuring the mental workload induced by the use of a wearable assistive device were included in this study. Three main families of methods were identified, the most common being ’dual task’ and ’subjective assessment’ methods, followed by those based on ’physiological measures’, which included a wide variety of methods. The variability of the measurements was particularly high, making comparison difficult. There is as yet no evidence that any particular method of measuring mental workload is more appropriate to the field of wearable assistive devices. Each method has intrinsic limitations such as subjectivity, imprecision, robustness or complexity of implementation or interpretation. A promising metric seems to be the measurement of brain activity, as it is the only method that is directly related to mental workload. Finally, regardless of the measurement method chosen, special attention should be paid to the measurement of mental workload in the context of wearable assistive devices. In particular, certain practical considerations, such as ecological situations and environments or the level of expertise of the participants tested, may be essential to ensure the validity of the mental workload assessed.

## Introduction

Wearable assistive devices aim to improve the mobility of their users, either by preserving or enhancing the motor performance of able-bodied people or by restoring the motor abilities of disabled people. Advances in mechatronics and robotics over the past few years have allowed for a significant acceleration in the development of increasingly sophisticated and efficient assistive devices [1]. However, a major challenge remains and tends to worsen due to the increasing complexity of these tools: the mental workload associated with using these devices [2, 3]. Mental workload was already identified as a critical criterion for acceptance of assistive devices in the 1970s and the rise of myoelectric prostheses [4], but it is still possible that it will increase with the growing complexity of the devices.

Although quite intuitive and widely used, the concept of mental workload comes with a plurality of definitions [5], which depend mainly on the context of the study. Globally, it can be described as “how hard the brain is working to meet task demands” [6]. In a human-computer interaction context such as that of wearable assistive devices, mental workload can be considered as the “demand placed on the user by the system” [7]. However, the mental workload depends on many parameters, notably on the intrinsic difficulty of the task performed but also on the subjective experience of the user. Thus, for the same task, two people will not have the same mental workload, depending on their initial capacities, their experience, their reaction to time pressure or fatigue, etc.

Depending on the scenario and the user’s cognitive abilities, the mental workload induced by the use of an assistive device may be such that the user may be unable to perform a parallel task or fully exploit the capabilities of the device, which could eventually lead to its abandonment [8]. For example, a robotic rehabilitation device that is too complex to use could negatively impact the patient’s motivation [9] and limit his or her involvement in the recovery process [10], greatly affecting the overall beneficial therapeutic effect [11]. Upper limb prosthesis wearers tend to compensate for the slowness and cumbersomeness of prosthesis control by relying on compensatory body movements [12], and may end up using the motorized prosthesis as a mere rigid tool or replacing it with overuse of their intact limb [13]. While walking is considered an activity requiring little or no cognitive effort for an able-bodied person, the use of a lower limb prosthesis or exoskeleton requires a high level of concentration [14], preventing the user from walking on uneven ground at the risk of falling [15], or performing a second parallel task such as holding a conversation [16]. The use of an exoskeleton to enhance physical performance should also not hinder the wearer in performing tasks such as giving orders or reading a map in the case of a soldier [3].

The mental workload caused by the use of assistive devices thus becomes a major consideration for the ecological/realistic use of assistive devices and their long-term adoption. This will only be possible if the balance between the benefits and burdens of use is strongly in favor of using the device. It is therefore crucial to be able to assess the mental workload imposed by the use of assistive devices, to ensure that the devices designed generate a minimal mental workload so that they can actually be usable and used.

However, measuring mental workload is quite complex. Measurement methods are numerous and variable, and the current literature does not specify which methods are appropriate for measuring the mental workload associated with the use of a wearable assistive device. Although studies on mental workload measurement already exist [7, 17–19], they focus primarily on physiological measures and none are applied to the field of assistive robotics. Only [20] focused on wearable assistive robotics but only provided a quick comparison of mental workload measurement methods for electromyography-based prosthetic devices. In [21], the authors announce that they will publish different measures of cognitive performance during walking, applied to the use of lower limb prosthesis. So, as this topic is clearly gaining interest, the purpose of this review is to examine how mental workload related to the use of wearable assistive devices has been previously measured, to better understand their advantages and disadvantages, and to determine the most appropriate method based on the application.

## Overview

### Methodology

A literature search was conducted in major scientific databases (including PubMed, IEEEXplore, ScienceDirect, and Google Scholar), using combinations of the following keywords: *prosthesis, exoskeleton, orthosis* and *cognitive, mental* and *load, workload, effort, burden, demand, cost, strain*. An initial selection of articles was made, and their references were manually searched for additional articles. Based on these results, studies measuring the mental workload induced by the use of a wearable assistive device (real or simulated, with disabled or able-bodied subjects) were included in this review. Because this review focuses on different methods of measuring mental workload, a large number of articles simply mentioning the mental workload induced by an assistive wearable device without measuring it were excluded. In the end, 60 articles were selected for this analysis, all published before June 2021. Some articles using questionnaires as a measurement method (see below) may be missing from this selection, as it is difficult to find all studies using customized questionnaires or not mentioned in the abstract.

### Measurement methods

Techniques for measuring mental workload when using wearable assistive device applications can be classified in the same way as for other applications: subjective assessments, secondary task procedures, physiological measures, and modeling.

- Subjective assessments are based on questionnaires that allow the participant to assess his or her own mental workload through rating scales for items such as mental effort, fatigue, frustration, etc.
- Secondary task procedures are performance-based measures using a dual-task paradigm: the participant is asked to perform a secondary task simultaneously with the primary task. His or her performance on the secondary task then reflects the mental workload induced by the primary task.
- Various physiological measures are related to a person’s cognitive functioning and can therefore be used to measure mental workload. Five different types of measures are considered in this review: ocular activity, skin-based measures, cardiac activity, respiratory activity and brain activity.
- Cognitive modelling allows to estimate the complexity of a task and the time needed to perform it by breaking it down into small simulated mental steps. It can therefore be used to evaluate the mental workload induced by a task without having to actually perform it.

**Fig. 1:**
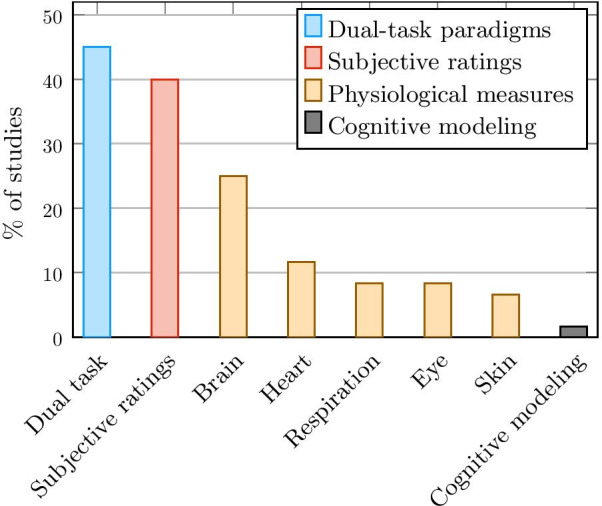
Distribution of mental workload measurement methods among reviewed assistive devices articles

Figure 1 presents the distribution of methods for the studies considered in this review. Subjective questionnaires and secondary task procedures are currently the two most widely used techniques for measuring mental workload in wearable assistive device applications (45% and 40% of studies, respectively). Among physiological measures, the measure of brain activity is the most investigated. In addition, various studies apply several measurement strategies in combination. In particular, when collecting physiological data, different parameters (cardiac activity, respiratory activity, etc.) are often investigated at the same time, and a questionnaire or a secondary task procedure can also be applied.

### Major facts

**Fig. 2:**
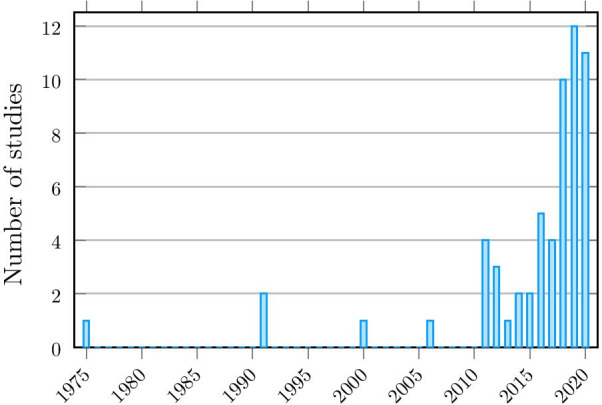
Distribution in time of the articles considered in this review, showing an acceleration of the publication on the subject

A growing number of articles mention that using a prosthesis is cognitively demanding (for example, for a Google Scholar search: *prosthesis “mental workload”* yields 1380 results, 1110 of which have been published since 2010, and 521 since 2018) as it becomes clear that despite advances in technology, the mental workload is not decreasing. However, only a small portion of these articles actually measured this mental workload. Figure 2 shows the chronological distribution of the studies considered in this review. It can be seen that although the number of articles per year is increasing, it remains low, with a maximum of 12 publications in 2019.

**Fig. 3:**
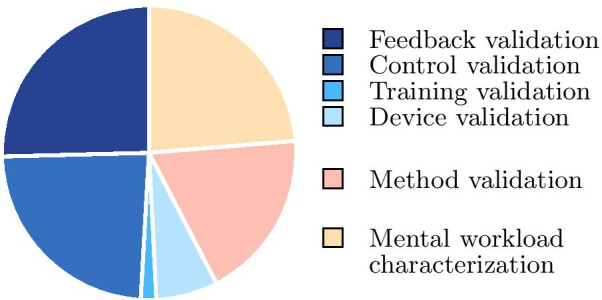
Distribution of the context of the articles considered in this review. In blue, studies using mental workload measurement as a tool to validate an innovation. In red, studies trying to validate a mental workload measurement method for the field of wearable assistive devices. In yellow, studies investigating mental workload itself

Figure 3 shows the distribution of the objectives of the studies considered. Many of the studies use mental workload measurement as a tool to compare or test the cognitive cost of using certain assistive technology innovations (shown in blue in Fig. 3). In particular, control modes, sensory feedback, training modalities and new device designs are evaluated. A second part of the papers attempts to validate a means of measuring mental workload for a wearable assistive device application (in red in Fig. 3). A third part deals with the study of mental workload itself (in yellow on the Fig. 3): their objective is to understand the cognitive difficulty of using a device (compared to healthy subjects for example), the possible adaptations to this cognitive difficulty, or the effects of a cognitive task simultaneous to the use of the device.

**Fig. 4:**
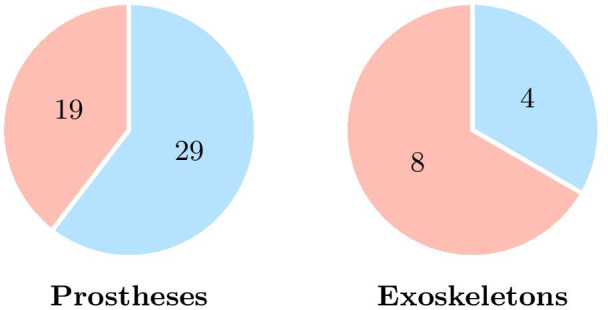
Distribution of upper limb devices studies (in blue) and lower limb devices studies (in red) among the considered studies on assistive devices

Of the selected studies, the largest proportion involved prosthetics (80%) compared to exoskeletons (20%). This is likely due to the fact that advanced bionic prostheses have been developed and available (even commercially) for more than a decade, whereas exoskeletons are rather new technological tools that are still in the development phase and have not yet faced usability issues such as mental workload. There are also slightly more studies on upper extremity devices (55%) than on lower extremity devices (45%). This difference is more pronounced for prostheses (60% for upper limb prostheses and 40% for lower limb prostheses) and can probably be explained by the major difference in the control between lower limb and upper limb systems. Indeed, lower limb prostheses typically offer discrete action control (i.e., a signal to be produced or a bodily motor behavior to be performed in order to activate a predefined assistance for a given task, such as standing or walking), whereas upper limb devices typically require users to continuously control the device at the joints. Controlling an upper limb prosthesis is therefore an important cause of increased mental workload and may explain the greater number of articles on upper limb prostheses. On the contrary, for exoskeletons, there are more studies for the lower limb than for the upper limb, which may be due to the greater number of lower limb exoskeletons developed to date. Figure 4 shows the distribution of upper limb device studies (in blue) and lower limb device studies (in red).

In the following section, the different types of methods used to assess mental workload when using portable assistive devices are described and analyzed. A table resuming the used mental workload measurements for all references can be found in Appendix.

## Mental workload measurement methods

### Subjective ratings

Although there are several standardized questionnaires for assessing mental workload (SWAT, RSME, etc.), the NASA-TLX (Task Load Index) [22, 23] is the only one used in the studies selected for this review. This questionnaire evaluates mental workload according to 6 aspects: mental demand, physical demand, perceived performance, temporal demand, effort, and frustration. Each item is rated by the subject using a bipolar scale giving a score between 0 and 100. A weighting procedure applied to each item then calculates the subject’s total mental workload or task load index [24–27]. However, an increasing number of studies use the raw workload index, which is the arithmetic mean of the 6 items [3, 28–37]. The independent analysis of the 6 subscales can also be used to differentiate between two tasks and determine which aspects of mental workload have the most impact on the subject [3, 29, 30, 32–34, 38–43].

Some researchers have added new subscales to the NASA-TLX to supplement it. For example, a “Conflicting Work Demand” item was added in [40], and an item regarding perceived embodiment of the prosthesis was added in [35]. Various similar questionnaires, but more specific to the field of wearable assistive robotics, were also introduced, either to replace the NASA-TLX [44, 45] or to complement it [35, 40].

Questionnaires (especially the NASA-TLX) have been widely used for many years and remain the standard. They are easy to implement and their reliability has been proven in various domains [23]; however, they remain subjective methods and therefore susceptible to bias by individual factors. For example, they depend heavily on the memory (in the case of extended protocols or multiple conditions, for instance) and self-analysis abilities of the participants, who can sometimes get lost between the perception of their own performance and the mental workload felt.

### Dual-task paradigms

Mental workload is related to performance, but is not the same thing. For example, an increase in mental workload does not necessarily imply a decrease in the subject’s performance if the subject is able to maintain his or her level of performance by increasing effort or changing strategy. However, the two concepts are related and this is why dual-task paradigms, which are performance-based measures, are among the most widely used methods for measuring mental workload. In a dual-task paradigm, the subject is asked to perform a second cognitively demanding or attention-demanding task in parallel with his or her primary task (which in our specific case is the use of a prosthesis or exoskeleton). If the mental processing of the second task conflicts with that of the first task, the performance on one or both tasks will be decreased in order to continue the execution of the tasks. It is then possible to compare the performance obtained for different task conditions: the lower the performance, the higher the mental workload associated with the task condition. Depending on the task on which the user is asked to concentrate, the performance of the primary task and/or the secondary task can be analyzed. Without trying to measure mental workload precisely, dual tasks are also used to have a more realistic context and/or to verify that the user is able to perform an action while using the device [3, 28, 46–50].

In the context of wearable assistive devices, different types of tests have been used. These are mainly mathematical tests (subtraction by 7 or 3, addition, etc.) [45, 46, 50–55], verbal fluency tests (vocabulary, spelling, etc. ) [45, 50, 51, 54, 56, 57] and memory tests [47, 58, 59]. Several studies also use approaches based on audio stimuli [3, 4, 52, 60, 61] or visual stimuli [3, 16, 33, 40, 52, 62]. However, these visual tasks are typically used with lower limb devices, as the use of upper limb devices already requires a high level of visual attention. For these devices, various secondary tasks that can be performed with the subject’s intact limb have been used [49, 63].

Dual-task paradigms may be of interest to the field of wearable assistive robotics, as cognitive and motor activities have been shown to be interdependent. In particular, there are interactions between walking and cognition: the addition of a second cognitive task disrupts the quality of walking, even in able-bodied individuals [64]. This is most noticeable in people with lower limb prostheses, who report a great need for concentration during walking. Thus, studies of lower limb prostheses using a dual task to measure mental workload typically examine variations in subjects’ gait in addition to cognitive task performance through several gait characteristics, such as walking speed, cadence, stride time, step length, sway, asymmetry [14, 16, 45, 51, 52, 65–67].

In conclusion, although dual-task paradigms are a popular method, approximately 25% of the studies selected for this review report non-significant differences in mental workload when measured with a dual-task paradigm. The reasons often cited for this lack of difference in mental workload are a too small difference in difficulty between the two main tasks that are compared, or a second task that is not challenging enough.

### Physiological measures

#### Ocular activity

Ocular activity monitoring provides several parameters that can be used to measure mental workload, such as pupil dilation, blink rate, gaze, etc. Among these different parameters, only pupil dilation, which corresponds to the variation in pupil size, has been used to estimate mental workload in the context of upper limb prosthesis applications. Indeed, pupil dilation has been shown to reflect mental effort and attention [68]. Larger pupil size tends to indicate more intense cognitive processing. This method has been particularly used to estimate mental workload for upper limb prosthesis applications. Using a Facelab eye-tracking system emitting infrared light, researchers in [69–71] measured pupil diameter as significantly larger when the prosthesis control mode was rated as more difficult by the user. With Tobii Pro2 eyeglasses, researchers in [72] also found a larger pupil diameter when the user experienced difficulty controlling their prosthetic hand.

In conclusion, pupillometric data appear to be a reliable metric for measuring mental workload. However, they must be used in a controlled environment, as the data can be corrupted due to changes in ambient light for example. Also, with a fixed eye-tracking system such as that of [69–71], the location of the subject relative to the system is very important, and data can be lost if he/she is misplaced or moves too much. This is probably why it has only been used for upper body devices and in laboratory environments, not in ecologically valid situations.

#### Skin-based measures

Electrodermal activity (EDA), also known as galvanic skin response, refers to the change in electrical properties of the skin in response to sympathetic nervous system activity on the sweat glands. The EDA measurement consists of two different components: skin conductance level (SCL), which corresponds to tonic changes in skin conductance, and skin conductance response (SCR), which corresponds to phasic changes in skin conductance measured by the number and intensity of spikes. SCL and SCR have been shown to increase with mental demand [73–75].

SCL increased when using a more mentally demanding interface to control an arm exoskeleton [38]. However, no significant effect of SCL was observed between two different modalities of prosthesis feedback [24, 25], or when introducing a cognitive task during walking with a lower limb prosthesis [33]. Regarding the SCR measure, the researchers of [33] extracted the frequency and amplitude of EDA spikes. However, neither measure changed significantly when adding a cognitively demanding task during walking with a lower limb prosthesis [33] or when using a hand prosthesis [25].

In conclusion, while skin measures have been shown to be well-established indicators of mental workload in general [76], only one of the studies in this review obtained a significant result using this type of measure [38]. This is likely due to the fact that skin measures are very sensitive to physical load and therefore not suitable for tasks requiring physical activity. For example, the physical effect of walking in [33] may have hidden the effect of mental workload on the EDA. Also, conductivity and skin temperature change quite slowly: it takes several minutes for them to change with the level of mental workload, which could explain the lack of results in [24, 25]. So, the influence of the physical activity and the slow electrodermal response, skin measurements may not be suitable for measuring mental workload when using assistive wearable devices.

#### Cardiac activity

The electrical activity of the heart is measured by electrocardiography (ECG), and several measures related to mental workload can be extracted, including heart rate (HR) and heart rate variability (HRV), the latter being a measure of the variation of time between each heartbeat. HR and HRV are well-established indicators of mental workload in a variety of domains because of the interactions between the autonomic nervous system and the cardiovascular system. Whereas an increase in HR is generally positively correlated with an increase in mental workload [73, 74, 77], a reduced HRV has been linked to poorer cognitive performance and higher cognitive demand [77, 78].

In [33], an increase in HR was observed both when a mentally demanding secondary task was introduced when walking with a lower limb prosthesis, and when the walking task was made more complex. It decreased when adding audio-visual feedback to an upper limb prosthesis [24, 25], suggesting that the added feedback reduces the cognitive demand of prosthesis use. However, no significant difference in mean HR was observed for an increase in prosthesis task speed [4], or when changing the control mode of an exoskeleton [38].

HRV can be analyzed both in the time domain and in the frequency domain. In both cases, a reduced HRV is related to a higher mental workload. In the time domain, the SDANN characteristic is the Standard Deviation of the Average instantaneous heart rate intervals (NN intervals) calculated over a moving window. It was significantly lower in those with difficulty using a BCI-controlled manual exoskeleton [39], and significantly lower for a more mentally demanding exoskeleton control mode [38]. Furthermore, it decreased over time in both control modes, suggesting increasing mental fatigue of participants as the experiment progressed. Similarly, the RMSSD feature, the square root of the mean squared differences of successive NN intervals, significantly decreased when a mentally demanding secondary task was introduced during walking with a lower limb prosthesis in [33]. In the frequency domain, reserchers in [24, 25] investigated changes in the 0.1 Hz component of HRV from a resting state, but no statistical difference was found. In [33], the ratio of low-frequency HRV components (0.04–0.15 Hz) to high-frequency HRV components (0.15–0.4 Hz) differed significantly between the walking condition, but not between with and without an added parallel cognitive task.

In conclusion, like skin measurements, HR and HRV are related to mental workload, but they are also highly sensitive to physical load and may not be the most appropriate measurements for wearable assistive device applications that typically require physical activity.

#### Respiratory measures

Respiratory measurements can include a variety of measures. Among these, respiratory rate, or breathing frequency, has been found to increase with mental workload and stress [74]. Although respiratory rate can be measured by electro-physiological methods, it has primarily been measured from the change in chest circumference during breathing. Using this measurement technique, researchers in [33] found that respiratory rate was one of the most sensitive physiological parameters to mental workload. Indeed, respiratory rate increased significantly when an additional cognitively demanding task was added while walking with a lower limb prosthesis. However, in [38], the control mode of the exoskeleton had no influence on respiratory rate. Similarly, in [4], subjects’ respiratory rate did not change as a function of the difficulty of the grasping task, and in [25], respiratory rate did not allow for conclusions about the mental workload induced by different sensory feedback modes.

In conclusion, although respiratory rate is related to stress and mental workload, it is also strongly influenced by physical activity. In [33], the difference in physical demand between symmetric and asymmetric walking was sufficient to induce a significant difference in respiratory rate. Furthermore, patients requiring an assistive wearable device are often frail, and the use of the device represents a significant physical effort for them, strongly impacting their breathing. Thus, respiratory rate as a method for measuring mental workload may not be appropriate for wearable assistive device tasks.

#### Brain activity

The physiological measures presented above provide an indirect evaluation of mental workload. Access to brain activity could allow a direct assessment. To date, two methods have been used: electroencephalography (EEG) and functional near-infrared spectroscopy (fNIRS). fNIRS measures changes in the relative concentrations of oxygenated and deoxygenated hemoglobin in the cortex due to neuronal activity [79]. It has only recently been used to assess workload during prosthetic use. In [80, 81], fNIRS showed a higher level of brain activity in lower limb prosthesis users than in controls for the same walking task. The introduction of a secondary cognitive task resulted in increased brain activity in [81]. fNIRS also discriminated the use of a prosthetic hand with or without sensory feedback in [82]. EEG, which measures the electrical activity of the brain (mainly pyramidal neurons), has been widely used and we will go into more detail about this method. Different aspects of EEG signals can be evaluated, especially in the time and frequency domain as we will see below.

*Time domain* A first EEG approach is based on event-related potentials (ERPs), which are spikes in the temporal EEG signal, time-locked to discrete stimuli (auditory, visual, somatosensory...) to which the participant does not necessarily have to pay attention. The ERP amplitude is inversely proportional to the mental workload of the main task at hand: the higher the cognitive demand, the lower the ERP amplitude evoked by the stimulus, reflecting a reduced availability of mental resources for processing the auditory or visual stimulus [83]. ERP components are named with a letter representing their polarity (P for positive, N for negative) and a number that indicates their latency (in ms) between the stimulus and the peak. The P300 component (also often referred to as P3), which appears approximately 300 ms after a stimulus, is one of the most studied ERP components, although the P200 component and “late positive potentials” (LPP) have also been studied in relation to mental workload. These components are usually measured on electrodes located on the midline of the head (i.e., mainly, but not exclusively, on Pz and Cz).

Researchers in [44] measured ERPs on Pz to distinguish mental workload during an easy level of upper limb prosthesis control from that during a difficult level. The results showed an inverse relationship between mental workload and the amplitude of P200, P300, and LPP. Regarding the lower extremity, researchers in [84] provided a proof-of-concept protocol using the P300 to measure mental workload in a population of seated, standing, and treadmill walking control participants. The P300 was able to distinguish sitting from walking after 30 minutes of task execution. For lower extremity devices, the P300 suggests that walking with a prosthesis without sensory feedback requires a greater mental workload than with sensory feedback [85, 86]. In the study [41], the P300 component measured on Cz also showed a significant difference between performing a task in the sitting condition and when walking with a prosthesis. In the same study, other ERP components were found to be relevant for measuring mental workload in a more subtle way. Indeed, the late positive potential at the Pz electrode and the P200 component at Fz, Cz, and Pz showed significant differences between easy and difficult levels of the task, while the P200 at Cz differentiated an intermediate level between easy and difficult. These examples show that EEG can distinguish mental workload between different conditions without adding an active task unrelated to the experimental condition, making the assessment not only direct but also ecological.

*Frequency domain* A second approach uses the frequency characteristics of EEG signals. Brain waves are divided according to their frequency into several sub-bands: delta (1–4 Hz), theta (4–8 Hz), alpha (8–13 Hz), beta (13–30 Hz) and gamma (30–80 Hz). Different functions have been attributed to these frequency bands, some of which may be related to mental workload. For example, delta oscillations have been found to be dependent on the activity of motivational systems, alpha oscillations on attention and theta oscillations on emotional regulation [87]. It has also been suggested that frontal theta may be a mechanism of cognitive control [88]. Not surprisingly, studies have assessed mental workload by power, entropy, and connectivity between regions for these EEG frequency bands.

The beta band ($\sim$ 14–35 Hz) has not been shown to vary with workload during walking with a prosthesis [25]. In contrast, alpha band power ($\sim$ 8–13 Hz) has been shown to be inversely related to mental workload during control of a manual prosthesis [89]. Indeed, by manipulating the feedback modality, alpha power was shown to decrease more when the NASA-TLX mental workload score was higher [25]. Similarly, an overall reduction in alpha power was observed when participants used a prosthetic hand instead of their natural limb [90, 91]. When walking with a prosthesis, low ($\sim$ 8–10 Hz) and high ($\sim$ 11–13 Hz) alpha power decreased as cognitive-motor task demands increased (due to cognitive task difficulty and/or task condition) [41, 43]. Gamma band power ($\sim$ 36–44 Hz) also decreased in the face of more difficult walking [43]. Conversely, theta band power ($\sim$ 4–7 Hz) was positively correlated with mental workload, increasing in the frontal region as cognitive-motor task demand increased [41, 43]. As might be expected from these results, the theta to alpha power ratio shows changes in mental workload [92]. The power of the fronto-theta/parietal-alpha (FT/PA) ratio increased with task difficulty [41, 43], as did the power of the fronto-theta/frontal-alpha (FT/FA) ratio [43]. The statistical entropy of the alpha band seems also positively related to mental workload: a rise in the entropy is hypothesized to reflect a less organized and more complex way of functioning of the brain [93]. In [42, 94], it increased with the mental fatigue of the participant and the difficulty of the task, and increased slower when the interface of prosthesis control is adaptive to the mental state of the participant. Finally, diminution of high-alpha connectivity between Fz (motor planning) and T7 (verbal-analytical) regions of the brain has been linked to a less conscious, less explicit control of a prosthesis [90]. Conscious control processes require considerable cognitive resources and therefore contribute to increase the mental workload. Overall, these results show that the relationship between workload and EEG signals is extremely complex but promising.

In conclusion, measurements of brain activity using methods such as EEG or fNIRS are interesting because they seem, by definition, to be directly related to mental workload. This field is evolving rapidly and the literature on the subject (outside the field of portable assistive robotics) is vast and growing. EEG measurements appear to have been used successfully in several above mentioned studies where the participants walked with a lower limb prosthesis [41, 43, 84–86, 91]. The development of wireless devices offers the possibility of making measurements under more realistic conditions. However, measurement of brain activity is not easily affordable, as EEG and fNIRS equipment are generally expensive, and signal processing remains complex.

### Cognitive modeling

Cognitive modeling differs from previous methods in that it aims to predict user performance in a task without performing an experiment, but by modeling how a user would interact with the system. Various computational models have been developed since the 1980s, mainly for the human-computer interaction domain.

Only one study has used cognitive modeling to assess the mental workload associated with the use of a wearable assistive device: researchers in [71] used a model from the GOMS family (Goals, Operators, Methods and Selection rules) to compare two modes of control of an upper limb prosthesis. To do this, a task with the prosthesis was divided into elementary operations belonging to three different categories: perceptual (visual, auditory, etc.), motor (movement of the arm, etc.) and cognitive (memorization of information, choices, etc.). A duration is associated to each operation, which finally allows to estimate the total duration required for the task.

The task with the prosthesis was modeled twice: once for a conventional myoelectric control mode, and once for a pattern recognition control mode. The pattern recognition control mode was shown to require fewer cognitive and motor operations for the same task. This would predict that this control mode will be less demanding than the other for this task. Furthermore, although the times calculated with the GOMS model are considerably less than those actually required to complete the task, the relative times of the different steps are similar.

In conclusion, a GOMS model can be used to predict/compare the usability of a prosthesis and the associated mental workload for a specific task, and possibly improve the human-machine interface to reduce the workload. Unlike other methods that provide a more global estimate, cognitive modeling allows a more detailed study of the origin of the demand difference by studying the distribution of operators in the categories. However, it only considers an expert user and focuses more on the intrinsic difficulty of the task, and not on the subjective experience of the user, the latter being an important component of the mental workload.

## Discussion

Given the different articles reviewed, several analyses can be performed. In the following section, we first provide some general observations about the field, then discuss the general challenges of accuracy and robustness in quantifying mental workload, and finally we consider several practical considerations related to the specificity of the wearable assistive robotics field.

### A growing and unsolved research question

While the measurement of mental workload has been extensively studied for over 40 years [5], it is only recently that the field of wearable assistive device research has begun to focus on it. This recent interest coincides with an increase in both the technological advances of these devices and the inherent complexity of their control and use. Given the distribution of methods (shown in Fig. 1), the fairly large number of studies on the validation of new metrics (shown in Fig. 3), and reviews of the literature, it is evident that the measurement of mental workload for wearable assistive devices remains an unresolved challenge, and that there is not yet an effective, universally agreed-upon method. Indeed, while subjective assessments and dual-task paradigms are the most commonly used methods, they suffer from imprecision. Methods based on physiological measures do not seem to be an effective alternative for various reasons, as mentioned above. Although there seems to be a growing interest in measuring brain activity, it is the most complex physiological measure to assess and interpret. Yet, research in the latter area is a hot topic, and thus it is the measure most likely to evolve. It is also and above all the only measure that is directly related to mental workload. As for the cognitive modelling method, it has so far been applied to too few studies to be able to assess its value in this area.

### The limited precision and robustness in measuring the mental workload

*Robustness of physiological measurements* Mental workload measures based on physiological measures are fundamentally influenced by many factors, some of which are difficult to control. Indeed, apart from the direct measurement of brain activity, other physiological measures capture the reactions of the autonomic nervous system. Thus, even if the measures used are indeed related to mental workload, their variation may also reflect the variation of many other parameters (environmental or internal). There is therefore a significant risk of misinterpreting a physiological signal believing that a variation is due to mental workload when in fact it is due to another phenomenon.

Physiological measures are, for example, very sensitive to physical activity. So, although a priori not included in (or even opposed to) *mental* workload by definition, *physical* workload is an important variable to take into account when measuring mental workload. There is a risk of observing false positives in increases in mental workload (e.g., in [40]), the increase in heart rate is due to the squatting position and not to the mental workload) or on the contrary false negatives, the mental workload being hidden by the physical demand [33]. It is therefore crucial to carefully examine the effect of physical load, especially in the field of wearable assistive devices for which cognitive and motor actions are obviously often used as evaluation tasks.

*The influence of psychological factors.* Psychological variations are often considered part of mental workload, although the exact definition of mental workload and whether or not psychological load (such as the “emotional load”) should be included are still under discussion [95]. In the context of sport, psychological workload has been referred to as the set of psychological demands a person faces that are primarily and directly associated with their life in and out of sport [96]. The field of wearable assistive devices is different in many ways, but there are some commonalities, particularly the physical aspects and the need to succeed. Therefore, as in sports performance, the same task repeated twice by varying a single psychological parameter (e.g., family, social) may yield two different measures of mental workload, even if the objective difficulty of the task has not changed. The distinction between psychological workload and mental workload is not necessarily important, but it can become a concern when trying to determine the cause of increased mental workload. Indeed, a physiological measurement does not allow to identify which parameter creates a variation in mental workload, since only an overall measurement is obtained. On the other hand, a well-designed questionnaire could allow this, as the user should be able to differentiate between intrinsic difficulty and psychological factors. Cognitive modeling does not take into account any psychological aspects and focuses only on the intrinsic difficulty of the task.

*Limited coherence of results* A major difficulty in the analysis of existing work lies in the absence of a precise and unified definition of mental workload. Indeed, even if the global concept may seem simple, there is a multiplicity of phenomena considered with their associated metrics. As the aim of this review is not to discuss the definition of mental workload, we have considered it as an open concept, bringing together the many different variables used to characterize it. But the multiplicity of measures can lead to inconsistency between the different results obtained. Recognizing the difficulty of characterizing mental workload, many studies used multiple measurement methods at the same time to obtain a more reliable measure. In particular, many studies used a subjective questionnaire in addition to the objective measure to confirm the latter [25, 33, 38, 40–45]. There are also studies using multiple measures at the same time, including various physiological measures [25, 33, 38]. If the simultaneous use of several measures most often allows the refinement of the measurement, the results obtained can sometimes be inconsistent or contradictory, as has been found in several studies [25, 44]. Indeed, each measurement method accesses mental workload in a different way: the dual-task paradigm focuses rather on attention management issues, physiological measures (outside brain activity) on autonomic nervous system variations, etc. Their different origins and influences may therefore lead to different measures of mental workload, which makes it difficult to compare the different metrics within the same study.

Comparison of the results obtained by the different studies is even more difficult, if not impossible. In addition to the variability of the parameters, the experimental setup and protocols used to characterize mental workload reinforce the difficulty of comparing results between studies, and thus in the present cases, the performance of assistive devices. Even the NASA-TLX, which has been widely used since its inception in 1988, can hardly provide an absolute reference for comparing mental workload levels. This lack of consensus may be detrimental since only a local comparison with control conditions in a given protocol is possible. Finally, in some studies, the inter-subject variability itself (and sometimes the intra-subject variability when several measurements are made during the day, due to the influence of environmental factors) strongly hampers the validity of the conclusions drawn.

*Temporal aspects* In most of the studies included in this review, measured data are used to calculate a level of mental workload after experimentation, not in real time (except for some specific applications, such as an adaptive interface in [42]). Indeed, the data obtained with most of the measurement methods considered require significant offline post-processing. Since the purpose of these studies is mainly to compare mental workload levels in different situations, this is not necessarily a problem. Also, the fact that the data require post-processing steps does not necessarily prevent good temporal accuracy of the measurement. However, there are also many measures that provide low-frequency information (such as the slowly evolving EDA) and considering only the full record over the task may allow for relevant analysis. Other metrics, although recorded continuously, may need to be averaged over a period of time to provide a representative measure because they are not robust to physical load or environmental factors (e.g., heart rate, pupil dilation). So, at the end they only provide a global estimate of the mental workload of the task. Finally, subjective evaluations, by their very nature, can only provide data at a specific time, usually at the end of the task. To have an intermediate measure, one would have to interrupt the task and fill out a questionnaire, which can interfere with the correct execution of the task. Therefore, such a single measure of mental workload on a complete task gives a less detailed and probably less accurate assessment of mental workload than a continuous measure with high temporal accuracy. Such an overall estimate, however, does not provide a clear understanding or identification of the origins or underlying factors of mental workload.

### Practical considerations

*Setup complexity* In addition to subjective assessments, dual-task paradigms, and cognitive modeling, the use of physiological measures tends to complicate the experimental setup with additional sensors for participants to wear (which can cause interacting problems with the sensors that are used to interact with assistive devices, or simply interfere with users’ natural abilities). It also adds experimental steps to set them up (the EEG being particularly time consuming) and sometimes to calibrate them. In addition, the cost of some of the required equipment (EEG system, eye tracker, etc.) may be a major factor minimizing the generalization of these, especially for intangible methods. Finally, some of the physiological measures require specific technical expertise to be processed and analyzed, which may limit their adoption outside specific research institutions. All of these aspects constitute a real barrier to the generalization of these methods.

*Ecological measurements* As observed, some parameters and protocols require standardized environments or controlled experimental conditions to avoid the influence of external variables (such as variation in light for pupil dilation, unexpected situations causing additional stress, an imposed strategy for performing a task under different conditions, or uncalibrated objects used in a task resulting in a variation in physical load). The assessment performed may not be fully representative of the actual use of the assistive devices. This is true for most approaches, because while this is more of an issue for physiological measures, even subjective assessments require standardized tasks and protocols to perform comparative score analyses.

*Naive or expert users* The mental workload of naive participants learning to use a wearable assistive device is different from that measured after some time of use, once they have become experts in the use of the device. Therefore, it is crucial to take into account the level of expertise of the participants in the experiment. Indeed, with users who are becoming familiar with a device, what is being measured may not be the mental workload induced by using the assistive device but rather by discovering and learning how to use it. One device may be difficult to grasp but easy to use once learned, while another moderately complex device may be easier to handle at first but will always be difficult to use. To account for this learning effect, studies could perform multiple measurements over different sessions, as [60] did over three days of experimentation. Also, since studies on measuring mental workload in the field are always a comparison between two devices (or modalities such as control mode, feedback, etc.), participants should have the same level of expertise in both modalities. However, it is difficult to organize a research experiment with participants who are experts in two different devices, especially with patients. For this reason, some teams try to assemble patients who are nearly naive to both devices and train them to have similar proficiency in both devices [54]. Other teams choose patients who have already experienced using their own assistive device and give them extensive training, up to several months, for another device [16, 45]. In any case, these aspects should definitely be taken into account to avoid any possible bias by confusing the level of expertise of the users with the difficulty of using a device when measuring mental workload.

## Conclusions

The field of wearable assistive devices is growing and becoming more mature, with research questions going beyond the technical aspects, including the ergonomics and usability of the devices. Consequently, there is a growing need for methods to measure and analyze the mental workload generated by these technologies. In this review, we summarized the methods used to assess the mental workload generated by the use of a wearable assistive device in 60 publications. The variability of the measures was particularly large, making their comparison difficult. Three main families of methods were identified. Subjective assessments and dual-task paradigms are the most commonly used methods, but they suffer from imprecision. Methods based on physiological measures, encompassing a wide variety of metrics, are also widely used for their objective dimension, but they are not very robust and therefore do not offer an optimal alternative. The measurement of brain activity, which is directly related to mental workload, is of growing interest in the literature and is the most likely to evolve profoundly. There is thus no consensus yet on a particular method of measuring mental workload that would be the most suitable for the field of wearable assistive devices. Regardless of the method chosen, several practical aspects must be carefully considered in this field, such as the impact of physical activity, psychological aspects, the level of expertise of the subjects and the ecological validity of the measurements. Ultimately, it is clear that the path to developing reliable measures requires a more fundamental understanding of mental workload, its definition, processes and influencing factors.

## Data Availability

Not applicable.

## Appendix

See Table 1.

**Table 1:** Summary of articles reviewed and associated methods for measuring mental workload

References	Authors	Year	Device	Limb	Mental workload measurement methods							
					Subjective rating	Dual-task paradigm	Physiological measures					Cognitive modeling
							Ocular	Skin-based	Cardiac	Respiratory	Brain	
[3]	Bequette et al.	2020	Exoskeleton	Lower	$\checkmark$	$\checkmark$						
[4]	Stassen et al.	1975	Prosthesis	Upper		$\checkmark$			$\checkmark$	$\checkmark$		
[14]	Morgan et al.	2016	Prosthesis	Lower		$\checkmark$						
[16]	Heller et al.	2000	Prosthesis	Lower		$\checkmark$						
[24]	Gonzalez et al.	2011	Prosthesis	Upper	$\checkmark$			$\checkmark$	$\checkmark$	$\checkmark$	$\checkmark$	
[25]	Gonzalez et al.	2012	Prosthesis	Upper	$\checkmark$			$\checkmark$	$\checkmark$	$\checkmark$	$\checkmark$	
[26]	Guggenberger et al.	2020	Exoskeleton	Upper	$\checkmark$							
[27]	Zhang et al.	2015	Prosthesis	Upper	$\checkmark$							
[28]	Bequette et al.	2018	Exoskeleton	Lower	$\checkmark$	$\checkmark$						
[29]	Connan et al.	2016	Prosthesis	Upper	$\checkmark$							
[30]	Crea et al.	2017	Prosthesis	Lower	$\checkmark$							
[31]	Karacan et al.	2020	Exoskeleton	Lower	$\checkmark$							
[32]	Kinne et al.	2020	Exoskeleton	Lower	$\checkmark$							
[33]	Knaepen et al.	2015	Prosthesis	Lower	$\checkmark$	$\checkmark$		$\checkmark$	$\checkmark$	$\checkmark$		
[34]	Lambelet et al.	2020	Exoskeleton	Upper	$\checkmark$							
[35]	Markovic et al.	2018	Prosthesis	Upper	$\checkmark$							
[36]	Olsen et al.	2019	Prosthesis	Upper	$\checkmark$							
[37]	Volkmar et al.	2019	Prosthesis	Upper	$\checkmark$							
[38]	Badesa et al.	2019	Exoskeleton	Upper	$\checkmark$			$\checkmark$	$\checkmark$	$\checkmark$		
[39]	Badesa et al.	2020	Exoskeleton	Upper	$\checkmark$				$\checkmark$			
[40]	Bridger et al.	2018	Exoskeleton	Lower	$\checkmark$	$\checkmark$			$\checkmark$			
[41]	Pruziner et al.	2019	Prosthesis	Lower	$\checkmark$						$\checkmark$	
[42]	Rezazadeh et al.	2012	Prosthesis	Upper	$\checkmark$						$\checkmark$	
[43]	Shaw et al.	2019	Prosthesis	Lower	$\checkmark$						$\checkmark$	
[44]	Deeny et al.	2014	Prosthesis	Upper	$\checkmark$						$\checkmark$	
[45]	Williams et al.	2006	Prosthesis	Lower	$\checkmark$	$\checkmark$						
[46]	Fasola et al.	2019	Exoskeleton	Lower		$\checkmark$						
[47]	Foldes et al.	2013	Prosthesis	Upper		$\checkmark$						
[48]	Neuhaus et al.	2011	Exoskeleton	Lower		$\checkmark$						
[49]	Raveh et al.	2018	Prosthesis	Upper		$\checkmark$						
[50]	Sharma et al.	2016	Prosthesis	Lower		$\checkmark$						
[51]	Howard et al.	2017	Prosthesis	Lower		$\checkmark$						
[52]	Morgan et al.	2017	Prosthesis	Lower		$\checkmark$						
[53]	Phillips et al.	1991	Exoskeleton	Lower		$\checkmark$						
[54]	Resnik et al.	2018	Prosthesis	Upper		$\checkmark$						
[55]	Yamada et al.	2016	Prosthesis	Upper		$\checkmark$						
[56]	D’Anna et al.	2019	Prosthesis	Upper		$\checkmark$						
[57]	Matulevich et al.	2014	Prosthesis	Upper		$\checkmark$						
[58]	Stepp et al.	2012	Prosthesis	Upper		$\checkmark$						
[59]	Valle et al.	2020	Prosthesis	Upper		$\checkmark$						
[60]	Aboseria et al.	2018	Prosthesis	Upper		$\checkmark$						
[61]	Brown et al.	2011	Prosthesis	Upper		$\checkmark$						
[62]	Geurts et al.	1991	Prosthesis	Lower		$\checkmark$						
[63]	Ray et al.	2018	Prosthesis	Upper		$\checkmark$						
[65]	Hunter et al.	2018	Prosthesis	Lower		$\checkmark$						
[66]	Hunter et al.	2018	Prosthesis	Lower		$\checkmark$						
[67]	Frengopoulos et al.	2018	Prosthesis	Lower		$\checkmark$						
[69]	Zhang et al.	2016	Prosthesis	Upper			$\checkmark$					
[70]	White et al.	2017	Prosthesis	Upper			$\checkmark$					
[71]	Zahabi et al.	2019	Prosthesis	Upper			$\checkmark$					$\checkmark$
[72]	Lindner et al.	2020	Prosthesis	Upper			$\checkmark$					
[80]	Moller et al.	2019	Prosthesis	Lower							$\checkmark$	
[81]	Moller et al.	2020	Prosthesis	Lower							$\checkmark$	
[82]	Thomas et al.	2021	Prosthesis	Upper							$\checkmark$	
[84]	Swerdloff et al.	2020	Prosthesis	Lower							$\checkmark$	
[85]	Petrini et al.	2019	Prosthesis	Lower							$\checkmark$	
[86]	Petrini et al.	2019	Prosthesis	Lower							$\checkmark$	
[90]	Parr et al.	2019	Prosthesis	Upper							$\checkmark$	
[91]	Ortiz et al.	2020	Prosthesis	Upper							$\checkmark$	
[94]	Rezazadeh et al.	2011	Prosthesis	Upper							$\checkmark$	

## Abbreviations

BCI: Brain-computer interface
ECG: Electrocardiography
EDA: Electrodermal activity
EEG: Electroencephalography
ERP: Event-related potential
fNIRS: Functional near-infrared spectroscopy
GOMS: Goals, operators, methods and selection rules
GSR: Galvanic skin response
HR: Heart rate
HRV: Heart rate variability
NASA-TLX: NASA task load index
RMSSD: Root mean square of successive differences
RSME: Rating scale mental effort
SCL: Skin conductance level
SCR: Skin conductance response
SDANN: Standard deviation of the average instantaneous heart rate intervals
SWAT: Subjective workload assessment technique.
